# Both piRNA and siRNA Pathways Are Silencing Transcripts of the *Suffix* Element in the *Drosophila melanogaster* Germline and Somatic Cells

**DOI:** 10.1371/journal.pone.0021882

**Published:** 2011-07-14

**Authors:** Nickolai A. Tchurikov, Olga V. Kretova

**Affiliations:** Department of Genome Organization, Engelhardt Institute of Molecular Biology, Russian Academy of Sciences, Moscow, Russia; Virginia Tech, United States of America

## Abstract

In the *Drosophila melanogaster* germline, the piRNA pathway silences retrotransposons as well as other transcribed repetitive elements. *Suffix* is an unusual short retroelement that was identified both as an actively transcribed repetitive element and also as an element at the 3′ ends of the *Drosophila* non-LTR F element. The copies of *suffix* that are F element-independent are far more actively transcribed than their counterparts on the F element. We studied the patterns of small RNAs targeting both strands of *suffix* in *Drosophila* ovaries using an RNase protection assay and the analysis of the corresponding RNA sequences from the libraries of total small RNAs. Our results indicate that *suffix* sense and antisense transcripts are targeted mainly by 23–29 nucleotides in length piRNAs and also by 21 nucleotides in length siRNAs. *Suffix* sense transcripts actively form longer RNA species, corresponding either to partial digestion products of the RNAi and Piwi pathways or to another RNA silencing mechanism. Both sense and antisense *suffix* transcripts accumulated in the ovaries of homozygous *spn-E, piwi* and *aub* mutants. These results provide evidence that *suffix* sense and antisense transcripts in the germ line and soma are targeted by both RNAi and Piwi pathways and that a Dicer-independent pathway of biogenesis of siRNAs could exist in *Drosophila* cells.

## Introduction

There are three distinct RNA-silencing pathways in *Drosophila*. The first is RNA interference (RNAi), which acts via 21-nucleotide (nt)-long siRNAs that originate from endogeneous long double-stranded RNAs (dsRNAs) and silence mRNAs from retrotransposable elements and host genes [1]–[3]. The second pathway is the microRNA (miRNA) pathway, in which small RNAs inhibit mRNA translation [4]. Finally, the third RNA-silencing pathway is the Piwi pathway, in which longer, 24–29 nt, piRNAs silence retrotransposons and other transcribed repeated elements in the germline [5]–[12]. piRNAs can both activate and repress transcription [13].

Normal accumulation of somatic endogeneous siRNAs that are complementary to mRNAs requires both Dicer-2, an endoribonuclease that generates siRNAs from long dsRNA, and the RNAi effector protein Ago2 [3], [14]. In *Drosophila,* miRNAs that are partially complementary to mRNAs are generated by Dicer-1, which acts with a dsRNA-binding protein partner termed Loquacious [15]–[18]. The Piwi pathway of RNA-silencing in *Drosophila* requires members of the Piwi subfamily of Argonaute proteins, including Piwi, Aubergine (Aub) and Ago3 [7]–[10]. piRNAs, which are predominantly antisense to retrotransposons and transposons, bind to Piwi and Aub proteins and guide the generation of sense piRNAs by cleaving sense retrotransposon transcripts [9], [10]. The Ago3 protein binds to sense piRNAs and can cleave the long antisense transcripts produced by clusters of different retrotransposons [9], [19]. Brennecke et al., 2007, suggested that the piRNA precursor (primary piRNA) is a long, single-stranded transcript. Recently a specialized piRNA pathway acting in germline and somatic tissues of the *Drosophila* ovary was described [11].

Recently, new research on piRNAs has drawn fresh attention to the clusters of *Drosophila* mobile elements found in heterochromatin. Such clusters were originally detected many years ago using molecular techniques, but their role was not well understood [20], [21]. Later, genetic techniques implicated *flamenco*, an element found in heterochromatin on the X chromosome, in the transposition regulation of several mobile elements [22], [23]. Complete sequencing of the *D. melanogaster* genome revealed that a small number of clusters of mobile elements or their fragments were trigger loci that produced piRNAs in the germline that repressed many retrotransposons [10], [19]. These piRNAs are amplified through reciprocal cycles of cleavage (ping-pong) by the Piwi/Aub and Ago3 proteins [10], and these cycles are germ-cell specific [11]. The data further indicated that *flamenco* is the source of piRNAs that target several types of retrotransposons and that *flamenco*-derived piRNAs almost exclusively occupy Piwi complexes [11].

It was demonstrated recently in *Drosophila* that endogeneous siRNAs derived from transposons are generated in somatic cells, while transposon transcripts are cleaved by the Piwi pathway in the germline [3]. It was also described that endogeneous siRNAs in *Drosophila* are targeting both protein-coding genes and mobile elements in both gonadal and somatic tissues [14]. *Suffix* is an unusual short retroelement in that there are separate conserved copies of the element, as well as divergent copies, in the 3′ untranslated regions of three genes [24]. *Suffix* has also been identified in the opposite polarity on the 3′ end of the *Drosophila* non-LTR F element, where it forms the 8^th^ conserved domain of a reverse transcriptase [25], [26]. Fragments of *suffix*, together with fragments of other retrotransposons, have also been detected in genomic DNA [27]. There are additional copies of *suffix* inside microsatellite regions consisting of (CAACA)_n_ repeats [28]. Transcripts from both strands of *suffix* have been detected at all stages of *Drosophila* development, with both *suffix*-specific siRNAs and longer piRNAs detected in the ovaries [2]. *Suffix-*specific RNAi leads to silencing of the relative LINE (long interspersed nuclear element) F element, suggesting that SINE-specific RNAi could downregulate genes with SINE stretches in their 5′ or 3′ non-coding regions (a phenomenon known as concerted silencing) [2], [29], [30].

The aim of the present study was to study small RNAs targeting *suffix* sense and antisense transcripts in the ovaries of wild-type flies and in a number of homozygous mutants. We showed that in *Drosophila* ovaries and somatic cells there is a class of short *ago3*-dependent piRNAs with the length of 23–29 nt, and that *suffix* sense and antisense transcripts are silenced by both the RNAi and Piwi pathways. In *aub*, *piwi* and *spn-E* homozygous mutants, both sense and antisense *suffix* transcripts accumulated at high levels, which independently confirmed that the RNAi and Piwi pathways are involved in cleaving *suffix* transcripts. Because *suffix* is actively transcribed into longer transcripts, including mRNAs containing the antisense strand of the element, we surmise that sense *suffix*-specific 23–28-nt piRNAs may be involved in efficient silencing of *suffix*-containing mRNAs. In this scenario, *suffix* could act as a “label” for transcripts that are designated for “concerted silencing,” a suggested mechanism that uses dispersed SINEs sequences in non-coding regions of different mRNAs as targets for RNAi-mediated synchronous silencing of SINE-containing genes [2].

## Results

### *Suffix* antisense transcripts are silenced in *Drosophila* ovaries and somatic cells mainly by formation of 23–27-nt piRNAs and 21-nt siRNAs

**Figure 1:**
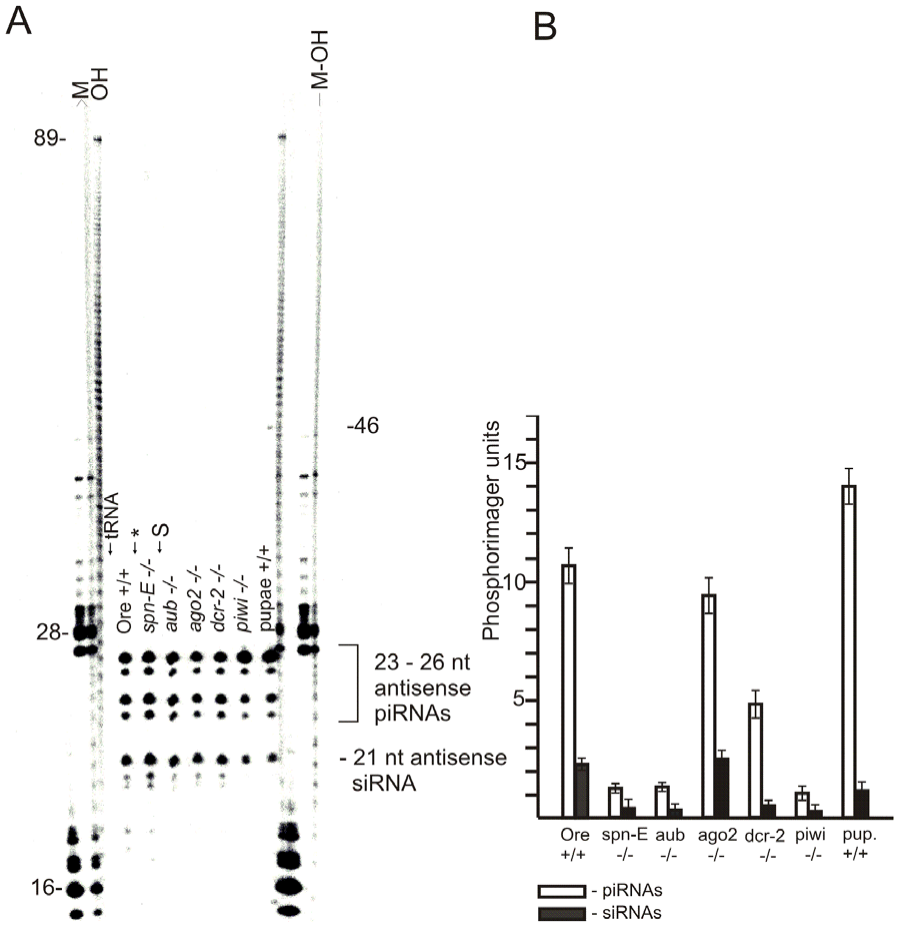
Visualization of *suffix* antisense small RNAs from *Drosophila* ovaries by a RNase protection assay using the sense RNA probe and separation on a high-resolution denaturing acrylamide gel. (A) A [^32^P]-labeled gel-purified sense RNA probe corresponding to the 5′ region of *suffix* was hybridized overnight with about 2–5 µg of total RNA isolated from the ovaries of Oregon R wild-type flies (Ore) or homozygous mutant flies (*spn-E*, *aub*, *ago2*, *dcr-2* and *piwi*), with total RNA from wild-type pupae or with 5 µg of yeast tRNA. The asterisk indicates results obtained using 5 µg of total *Drosophila* RNA without hybridization (overnight incubation at 0°C). S – self-annealing of the probe alone, without any RNA (between *spn-E* and *aub* lanes). M – RNA markers, corresponding to RNA synthesized by T7 RNA polymerase on pGEM-1 plasmid templates digested by *EcoR*I or *Sma*I enzymes. OH – a partial base-hydrolysis ladder of the gel-purified [^32^P]-labeled sense RNA probe. M-OH – a partial base-hydrolysis ladder of the gel-purified [^32^P]-labeled sense RNA probe mixed with RNA synthesized by T7 RNA polymerase on pGEM-1 plasmid template digested by *Sma*I enzyme. The lengths of the RNA molecules (in nt) are as indicated. Antisense *suffix* siRNAs and piRNAs are indicated by the dash and the bracket, respectively. (B) Quantification of the separation data. The data shown in panel A were normalized using rp49 as an internal reference. Error bars represent the results obtained in four independent experiments.

*Suffix* is actively transcribed during all stages of *Drosophila* development, and both sense and antisense RNA transcripts are found in somatic cells as well as in ovaries and testis [2]. We used an RNase protection assay to visualize small RNAs derived from *suffix* transcripts. In preliminary experiments, we optimized conditions for the complete digestion of the gel-purified strand-specific [^32^P]-labeled *suffix* transcripts. No protection of the labeled RNA probes was observed in self-annealing or tRNA-annealing experiments (Figure 1A). In contrast, when total RNA was annealed with the [^32^P]-labeled *suffix* sense transcript, followed by RNase treatment, we observed 21 nt band corresponding to *suffix* antisense siRNAs and 23–26 nt bands corresponding to *suffix* antisense piRNAs (Figure 1A). Quantitation of the phosphorimager data indicated that in ovaries of Oregon R wild-type flies, about 20% of the label corresponded to siRNAs and 80% to piRNAs. To validate the RNase protection assay we used 5′ phosphorylated synthetic RNAs of different length corresponding to *suffix* sense strand. The data shown in Figure S3 indicate that the assay used can provide a correct estimation of unlabeled RNAs length.

The RNase protection assay was performed using total RNA isolated from the ovaries of Oregon R wild-type flies as well as from a number of homozygous mutant fly lines: *spn-E^1^*, *aub*, *ago2*, *dcr-2* and *piwi*. RNase protection experiments showed that these RNAs and RNA isolated from wild-type Oregon R pupae had the same pattern of *suffix* antisense small RNAs, with the exception of a 46-nt RNA band present only in the pupae sample. In all other RNA samples, longer RNA species were not detected in these experiments, which indicates that *suffix* antisense transcripts were silenced by the siRNA and piRNA pathways in both *Drosophila* germline and somatic cells.

The phosphorimager data were normalized using rp49 cDNAs prepared from the same total RNA preparations as an internal reference. In both wild-type and mutant ovaries, *suffix* antisense piRNAs were more abundant than siRNAs (Figure 1B). However, the ratio between these small RNA species and their amounts varied among different mutant ovaries. Lower levels of piRNAs were observed in *spn-E^1^*, *aub*, and *piwi* homozygous mutants. A high piRNA/siRNA ratio was observed in the homozygous *dcr-2* mutant due to a ∼5-fold reduction of the amount of siRNA. The well-reproduced data on persistence of *suffix* antisense 21 nt siRNAs in the mutant clearly suggest the existence of a Dicer-independent mechanism of dsRNA cleavage that could co-exist with the Dicer-dependent pathway or could be activated in *Drosophila* cells only when the dependent pathway is damaged.

We observed no partial digestion products that could potentially have formed during dicing of long dsRNAs. The detected antisense 21–26-nt RNAs probably are designated for targeting of *suffix* sense transcripts. Analysis of *suffix* antisense RNAs in libraries of small RNAs (see below) also revealed that the major part of the observed 23–26-nt *suffix* antisense RNA species belong to a piRNAs class and only a small part corresponds to siRNAs.

### *Suffix* sense transcripts are silenced in *Drosophila* ovaries and somatic cells by formation of siRNAs, piRNAs and longer RNA species

**Figure 2:**
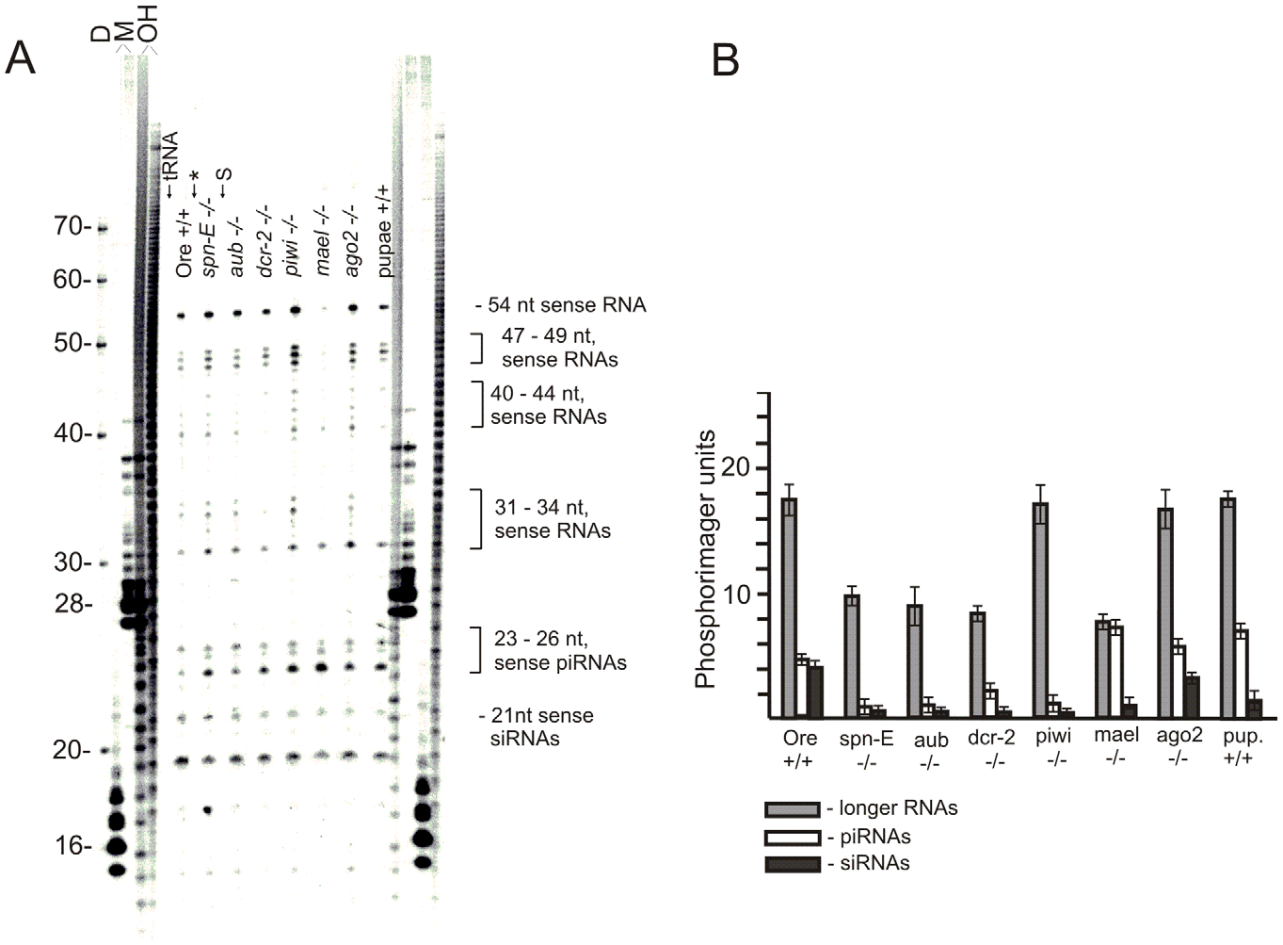
Visualization of a pattern of *suffix* sense small RNAs from *Drosophila* ovaries by hybridization with the antisense RNA probe and separation on a high-resolution denaturing acrylamide gel after a nuclease protection assay. (A) [^32^P]-labeled gel-purified antisense RNA probe corresponding to the 5′ region of *suffix* was hybridized overnight with 2–5 µg of total RNA isolated from the ovaries of Oregon R wild-type flies (Ore) or homozygous mutant flies (*spn-E*, *aub*, *dcr-2*, *piwi, mael* and *ago2*), with total RNA from pupae or with 5 µg of yeast tRNA. The asterisk indicates results obtained using 5 µg of total *Drosophila* RNA without hybridization (overnight incubation with the probe at 0°C). S – self-annealing of the probe alone, without any RNA (between *spn-E* and *aub* lanes). D – decade RNA marker (Ambion). M – RNA markers, corresponding to RNA synthesized by T7 RNA polymerase on pGEM-1 plasmid templates digested by *EcoR*I or *Sma*I enzymes. OH – a partial base-hydrolysis ladder of the gel-purified [^32^P]-labeled sense RNA probe. The lengths of the RNA molecules (in nt) are as indicated. Sense *suffix* siRNAs, piRNAs and longer RNAs are indicated by the dash or brackets. (B) Quantification of the separation data. The data shown in panel A were normalized using rp49 as an internal reference. Error bars represent the results obtained in four independent experiments for longer RNAs, piRNAs, and siRNAs.

After annealing samples of total RNA with the [^32^P]-labeled *suffix* antisense probe and treating the reactions with RNase, we observed a series of RNA bands in the 19 to 54 nt region of the gel, reflecting a complex pattern of *suffix* sense small RNAs (Figure 2A). The RNase protection assay was performed using total RNA isolated from the ovaries of Oregon R wild-type flies and from a number of homozygous mutant fly lines: *spn-E^1^*, *aub*, *dcr-2, piwi*, *mael* and *ago2*. Similar band patterns were observed using all RNA preparations: 21 nt siRNAs and 23–26 nt piRNAs. Three groups of larger RNA bands were also observed: one group of bands was in the 31–34 nt region, two other groups were in the 40–44 nt and 47–49 nt regions, and an additional bright 54 nt band was also detected. Quantitation of the phosphorimager data indicated that about 7% of the label corresponded to siRNAs and 28% to piRNAs; the larger RNAs occupied about 65% of small RNAs spanning the 21–54 nt region. Stronger RNase treatment caused the bands in all regions to disappear (data not shown), suggesting that the larger bands were not the products of incomplete RNase digestion of the probe, but rather corresponded to hybrids with longer *suffix* sense small RNAs species. Our RNase protection experiments with the [^32^P]-labeled *suffix* sense probe independently suggested that in the conditions used the digestion of non-hybridized RNA probes was complete (Figure 1). RNA isolated from wild-type *Drosophila* pupae (Figure 2) contained the same small RNA bands as RNA isolated from ovaries from mutant fly lines.

The 31–54 nt bands (corresponding to *suffix* sense small RNAs) in Figure 2 might be partially processed products generated during the dicing of *suffix* dsRNAs and/or during primary piRNA slicing. However, the absence of partially digested Dicer products in these RNA preparations (Figure 1) strongly argues against the possibility that the longer RNA bands that appear above the piRNA bands belong to the RNAi pathway. Indeed, Dicer digests dsRNA, producing equal amounts of sense and antisense strands. That is why we conclude that the detected longer *suffix* sense RNAs do not correspond to intermediates of RNAi pathway.

The phosphorimager data shown in Figure 2A were normalized using rp49 cDNAs as an internal reference. In both wild-type and mutant ovaries, *suffix* sense piRNAs were more abundant than siRNAs (Figure 2B). However, the ratio between these small RNA species and their amounts varied in different mutant ovaries. The levels of *suffix* sense piRNAs in *spn-E^1^*, *aub*, and *piwi* homozygous mutants were lower and approached those of siRNAs in these probes. Although a ∼5-fold reduced amount of *suffix* sense siRNA in the *dcr-2* homozygous mutant was detected, the persistence of this band again suggests the existence of a Dicer-independent mechanism of dsRNA cleavage.

Our data also indicate that different approaches to quantifying small RNAs contents can produce differing results. Indeed, immunoprecipitation of small RNAs, RT-PCR analysis, selection of different classes of small RNAs for deep sequencing, or detection of small RNAs by Northern analysis or hybridization with microarrays each will identify a specific subset of the whole pool of particular small RNA sequences. Thus, we believe that the RNase protection assay also might select a subset of small RNAs that are more stable and survive under very strong RNase treatment. For this reason, we used other approached for independent estimation of the accumulation of small RNAs corresponding to both strands of *suffix*.

### Accumulation of *suffix* transcripts in *Drosophila* ovaries in homozygous and heterozygous mutant flies

**Figure 3:**
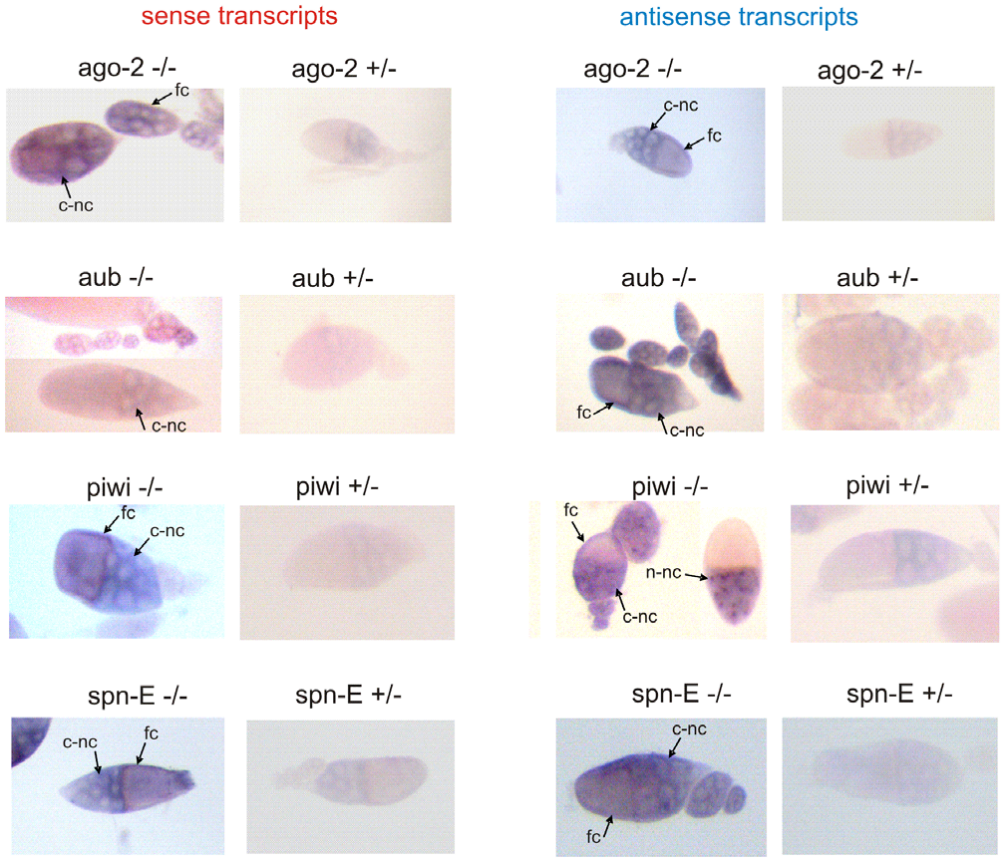
Accumulation of *suffix* transcripts in ovaries of homozygous and heterozygous mutants. *Suffix* sense and antisense transcripts in *Drosophila* ovaries were detected using *in situ* hybridization with DIG-labeled antisense and sense RNA probes, respectively. Arrows indicate the transcripts detected in the cytoplasm of nurse cells (c-nc), in nuclei of nurse cells (n-nc) and in follicle cells (fc).

We studied the effect of mutations in the *ago2*, *aub, piwi* and *spn-E,* genes on the accumulation of *suffix* transcripts in *Drosophila* ovaries. To visualize the transcripts, we used *in situ* hybridization with strand-specific DIG-labeled RNA probes using homozygous and heterozygous ovaries. In the mature egg chamber of the ovaries, which consists of the oocyte and nurse cells surrounded by somatically derived follicle cells, we observed that both sense and antisense *suffix* transcripts were present mainly in the cytoplasm of the nurse cells and follicle cells (Figure 3). In ovaries from the homozygous *ago2* −/−, *piwi* −/− and *spn-E* −/− mutants, the sense *suffix* transcripts, detected by hybridization of the DIG-labeled antisense probe, were clearly more abundant than in the corresponding heterozygous mutants. Levels of antisense transcripts, detected by hybridization with DIG-labeled sense probe, were also noticeably higher in the *spn-E* −/−, *aub* −/− and *piwi* −/− mutants. *spn-E* encodes a putative DExH-box RNA-helicase that is required for piRNA pathway silencing of *Drosophila* genomic repeats and retrotransposons [7], [31], [32]. We observed a dramatic accumulation of *suffix* transcripts in the *spn-E* homozygous mutant. These data suggest that the Spn-E helicase is an essential component of RNA-silencing machinery, although its precise function is not yet known. This enzyme is also important for silencing *suffix* transcripts. The *piwi* and *aub* genes are involved in *suffix* transcripts silencing, since when these genes are disrupted, *suffix* transcripts accumulate. In *aub* homozygous mutant ovaries considerable amount of *suffix* antisense transcripts were accumulated. Interestingly, in *ago2* and *aub* homozygous mutants, the higher levels of *suffix* sense transcripts were observed in both the late- and early-stage egg chambers. The same is true for the antisense *suffix* transcripts in *aub*, *piwi* and *spn-E* homozygous mutants. Only in *piwi* and *spn-E* homozygous mutants we observed accumulation of *suffix* antisense transcripts in both the cytoplasm and nuclei of the late- and early-stage egg chambers. These experiments were intended to generally localize the *suffix* transcripts in ovaries, and the transcript quantitation is just an estimate.

### The *aub*, *piwi* and *spn-E* genes are required for silencing *suffix* transcripts in *Drosophila* ovaries

**Figure 4:**
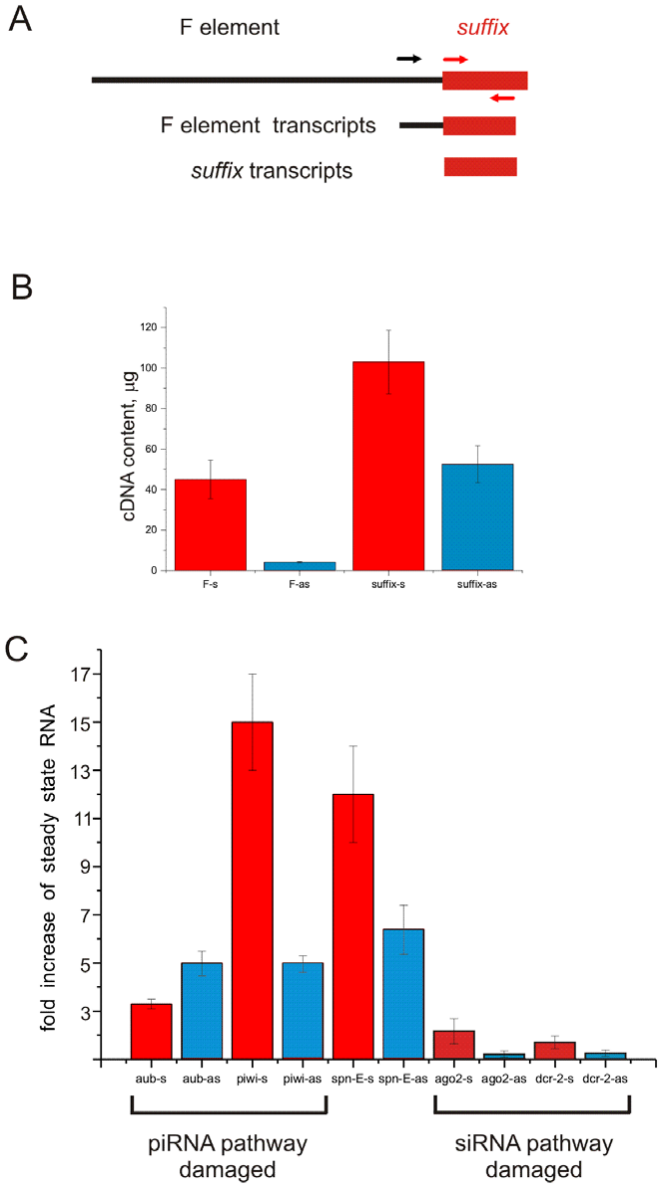
The levels of F element and *suffix* sense and antisense transcripts in Oregon R wild-type ovaries and accumulation of *suffix* transcripts in homozygous *aub*, *piwi*, *spn-E*, *ago2* and *dcr-2 Drosophila* ovaries. (A) A schematic presentation of relations between F element and *suffix* (not to scale) and primers used for RT-PCR to estimate F element and *suffix* transcripts. (B) Bars indicate amounts of sense and antisense transcripts corresponding to F element and *suffix*. (C) Bars indicate the ratio of *suffix* sense (red bars) or antisense (blue bars) transcripts in homozygous mutant fly ovaries compared to transcripts in the ovaries from wild-type flies. Transcript levels were normalized using rp49 mRNA levels. Error bars represent the data obtained in four parallel RT-PCR experiments.

We next used quantitative RT-PCR to estimate more precisely the accumulation of *suffix* transcripts in the ovaries of homozygous mutant flies. The data for sense and antisense transcripts of the *suffix* element in the wild-type stock and in the homozygous mutants were normalized using and rp49 mRNA levels. Sense *suffix* transcripts were more abundant than antisense transcripts in ovaries from the wild-type Oregon R stock (Figure 4A,B). Using a primer located in F element just upstream from *suffix* and the same minus primer inside the *suffix*, we also observed, that F element is transcribed less actively than *suffix* element. It follows that *suffix*-specific transcripts mainly arise from separate *suffix* copies.

In a previous study, a plot of piRNAs along the F element revealed the presence of numerous sense piRNAs that are loaded into Ago3 [10]. One of the regions with the most abundant piRNAs resides exactly at the 3′ end of the element, which corresponds to the *suffix* sequence. However, it was not recognized that these sense piRNAs mainly originate from separate copies of *suffix* that are more actively transcribed than the F element. The RT-PCR data shown in Figure 4B indicate that about 60% and 90% of *suffix* sense and antisense transcripts, respectively, origin not from F element, but from transcribed separate *suffix* copies. These RT-PCR data are in agreement with the earlier conclusion, based on the Northern hybridization data, according to which *suffix* is more actively transcribed than F element [2].

RT-PCR analysis also confirmed that *suffix* was derepressed in the ovaries of *aub*, *piwi* and *spn-E* homozygous mutant flies. Mutations in *aub*, *piwi* and *spn-E* caused an increase in both *suffix* sense and antisense transcripts (Figure 4C). Mutations in *aub*, *piwi* and *spn-E* resulted in 5-, 5.1-, and 6.2-fold increases, respectively, in antisense *suffix* transcripts. In the *aub* mutant, the sense transcript level only increased slightly (3.3-fold). However, in *piwi* and *spn-E* mutants, the increase was much higher: 15- and 12-fold, respectively.

In the RNase protection experiments, we observed both siRNAs and piRNAs derived from *suffix* sense transcripts. The RT-PCR experiments showed that in the absence of Aub or Piwi, which are critical proteins in the Piwi silencing pathway, *suffix* sense transcripts accumulate at high levels in the ovaries. The RNase protection experiments detected both 21-nt siRNAs (c 20%) and 23–26-nt piRNAs (c 80%) coming from *suffix* antisense strand (Figure 1). Using RT-PCR we observed an ∼5-fold increase of *suffix* antisense transcripts in the *piwi* and *aub* mutants, affecting piRNA pathway.

### Analysis of *suffix* small RNAs in libraries enriched for siRNAs and piRNAs

To elucidate the nature of 21–29-nt *suffix* antisense RNAs we used a study of *suffix* small RNAs in the sequenced libraries of small RNAs isolated from wild-type and mutant ovaries [12]. The data on frequencies of *suffix* small RNAs in ovaries isolated from the *ago3*/TM6B heterozygous and *ago3*/*ago3* homozygote are shown in Table 1. In the absence of *ago3*, the dramatic a 77 fold reduction of antisense *suffix* piRNAs and a 2.5 decrease in sense piRNAs were observed. The data strongly suggest that *ago3* is critical for generation of *suffix* antisense piRNAs. The data also indicate that Ago3 binds with *suffix* sense piRNAs and targets *suffix* antisense transcripts producing mainly 23–27-nt antisense piRNAs.

**Table 1:** Frequencies of suffix siRNAs and piRNAs in oxidized ovary data sets (normalized to total reads).

RNA type	Strand	Reads in ago3/TM6B	Reads in ago3/ago3	Fold decrease in ago3/ago3
siRNAs	sense	26.89	17	1.58
	antisense	132	46	2.87
piRNAs	sense	61.1	24	2.54
	antisense	1788.2	23	77.75

**Figure 5:**
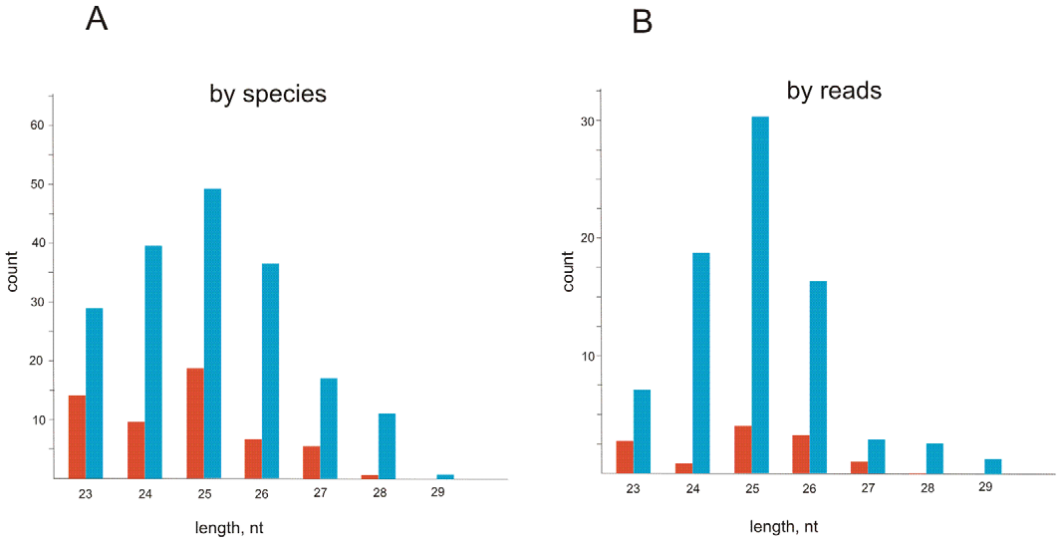
The length distribution of piRNAs for *suffix* in Oregon ovaries. The counts of 23–29 nt piRNAs in the small RNA library [12] are shown. Sense and antisense piRNAs are shown by the red and blue bars, respectively.

Analysis of sequences of *suffix* small RNAs from the libraries containing 23–29 nt stretches provide data on a spectrum of piRNA sequences and their frequencies. Figure 5 presents the length distribution of piRNAs for *suffix* in Oregon R ovaries by species and reads. *Suffix* antisense piRNAs are more diverse and abundant than sense piRNAs. The modal length for both antisense and sense piRNAs is 25 nt. RNase protection assays make these data directly visible as the patterns of fractionated RNA fragments with varying intensity. Our data obtained by these different approaches are in agreement; however, some differences are observed. The lengths of *suffix* sense piRNAs in RNase protection data are shifted to the lower values. Probably this is due to stronger RNase treatment that was used in this case in order to exclude the possibility of incomplete digestion of the probe and to be sure of the presence of longer RNA species separating above piRNAs. That is why 27–29-nt piRNAs were observed only on the overexposed gel shown on the Figure 2 (see also Figure S4).

**Figure 6:**
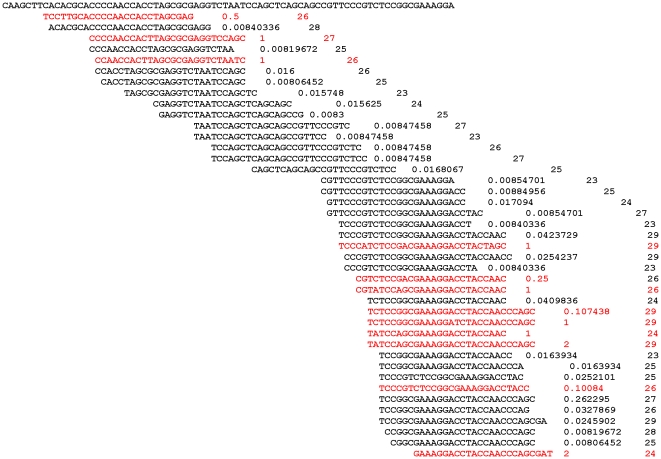
Alignment of *suffix* sense small RNAs from the 23–29 nt library of small RNAs from Oregon R ovaries [12]. The fragment of *suffix* shown on the top line corresponds to the fragment of the element used in RNase protection experiments (Figures 1 and 2). RNA species with frequencies above 0.1 are shown in red. The frequencies and the lengths of small RNAs are indicated.

There are several abundant piRNA size classes of *suffix* sense piRNAs in the range 24–29 nt corresponding to the region of *suffix* used in the RNase protection experiments (Figure 6). From these data it is clear that the smallest 19 nt RNA band on Figure 2 should correspond to the truncated rich piRNAs at the very end of the *suffix* fragment. The 3′ end of this fragment is highly “immunogenic” because it produces the greatest fraction of piRNAs species. These “nests” of piRNAs probably reflects the sequence preferences in the slicer-mediated mechanisms generating new piRNAs.

## Discussion

### Strand biases in *suffix* silencing via piRNA

*Drosophila* siRNAs are derived from both the sense and antisense strands of their double-stranded precursors, whereas piRNAs arise mainly from the antisense strand [7]. In the germline, 24–29-nt piRNAs are detected for most retrotransposons, suggesting that the piRNA pathway protects the fly germline from expressing selfish genetic elements [7], [8], [10]. When this paper was in preparation *Drosophila* germ line siRNAs and somatic piRNAs were also described [11], [14]. It is clear now that, like the majority of *Drosophila* retroelements, *suffix*-specific endogenous small RNAs in the germline are generated by both the siRNA and piRNA pathways. *Drosophila* ovaries contain the somatic follicular germline cells within the egg chamber. The whole pupa also contains both somatic and germline cells. For this reason, our RNase protection results alone cannot distinguish the source of *suffix* siRNAs or piRNAs. However, taken together with the *in situ* hybridization data on *piwi* −/− and *ago2* −/− mutants, which demonstrate the accumulation of *suffix* sense and antisense transcripts in both follicular and nurse cells (Figure 3), the results indicate that both siRNA and piRNA pathways silence both sense and antisense *suffix* transcripts in both somatic and germline cells.

The Piwi silencing mechanism uses primary piRNAs from piRNA cluster transcripts and maternally inherited piRNA complexes [10]. In these complexes, either sense or antisense 25–29-nt piRNAs are present, allowing slicer cleavage of long single-stranded piRNA cluster transcripts or long single-stranded retroelement transcripts. In this manner, very active expression of *suffix* sense transcripts is repressed during *Drosophila* development in wild-type lines [2].

How is the particular RNA strand selected by Ago3 or by the Piwi/Aub complexes? This is an interesting question, because 23–27-nt piRNAs arise mainly from the antisense strand for the majority of transposons. Ago3 predominantly binds to transposon sense transcripts [9], [10]. *ago3* is absolutely required for formation of *suffix* antisense piRNAs (Table 1). This fact suggests that Ago3 binds with *suffix* sense piRNAs and targets *suffix* antisense transcripts.

*Suffix* full-length sense transcripts were detected both in cultured cells and in pupae. *Suffix* sense transcripts (as part of longer RNAs) are extremely abundant during all stages of *Drosophila* development [2]. *Suffix* appears in opposite orientation on the 3′ ends of some genes, and the mRNAs of those genes have the *suffix* antisense sequence on their 3′ ends [24]. This is an example in which a gene's sense transcript and a transposon's antisense transcript are combined. It may be that strand selection occurs during the original transposon invasion, when Ago3 becomes the principal recipient of piRNAs from transposon mRNA; subsequently, this is preserved epigenetically via inheritance of maternal piRNA complexes.

*Suffix*-specific RNAi leads to silencing of the relative LINE – F element [2]. The presence of the *suffix* antisense sequence in genes could also lead to gene silencing. In this manner, a set of genes with a SINE in their 5′ or 3′ ends could also be silenced via concerted silencing [1], [29], [30]. This type of regulation, evolving from defense mechanisms, could be used to regulate genes by RNAi-related mechanisms during development.

### Clusters containing *suffix* sequences

Both sense and antisense *suffix* sequences can be found in very long polyadenylated transcripts in embryos and imagos [2].These transcripts probably correspond to clusters of mobile elements in the *Drosophila* genome [10], [20]. In the current version of the *Drosophila melanogaster* genome sequence, there are only a small number of separate copies of *suffix*. Most *suffix* copies listed in the fly databases are located on the 3′ ends of complete F elements. Full-length copies of this LINE are often surrounded by short fragments of mobile elements in random orientations. However, the separate *suffix* copies are transcribed far more actively than their counterparts in F elements [2].

We detected separate conserved and clustered diverged *suffix* copies inserted in a *Drosophila* satellite sequence in the form (CAACA)_n_ (work in progress). Between the clustered *suffix* copies, there are sometimes fragments of other mobile elements inserted in random polarities. This satellite sequence is located in heterochromatin on the right arm of chromosome 2 and on the Y chromosome [33]; it is ∼1 Mb, but is still absent from the most recent *Drosophila melanogaster* genome sequence. A number of other mobile elements in this satellite have been described previously [27], [33], [34]. It was demonstrated recently that *Drosophila* 1.688 satellite DNA is transcribed and that its transcription is regulated by RNAi [35]. It is not clear if the copies of *suffix* in the (CAACA)_n_ microsatellite regions are transcribed. In theory, they could give rise to the primary piRNAs and long transposon-containing transcripts.

Years ago, *Drosophila* mutants with increased transposable element mobilization of one particular or different transposable elements were described [36], [37], leading to the idea that in these mutants different elements that control transposition are affected in these mutants [38]. The data presented here raise the possibility that piRNA loci containing either one element or clusters of multiple mobile elements are affected in these mutants.

### Both *suffix* sense and antisense transcripts are targeted by both RNAi and Piwi pathways

Normal accumulation of endogenous siRNAs in *Drosophila* requires Dicer-2 ribonuclease and the RNAi effector protein Ago2. We observed that siRNAs were still present in *dcr-2* homozygous mutants. Nevertheless, our data also indicated that Dicer-2 was required for formation of *suffix*-specific siRNAs, because quantities of both *suffix* sense and antisense siRNAs in the ovaries of the homozygous *dcr-2*/*dcr-2* mutant were reduced as much as 5-fold compared with the wild-type ovaries (Figure 1B, Figure 2B). We thus suggest that another unknown ribonuclease could be involved in forming siRNAs in the fly RNAi pathway. In support of this hypothesis, a recent report noted that although there was a marked reduction in siRNA abundance in the *dcr-2^L811fsX^* null mutant, some endo-siRNAs still persisted [3]. These siRNAs were not detected previously in the RNase protection experiment using SI nuclease [2], which more easily removes shorter duplexes, than the mixture of RNases, that was used in the present study. Recently accumulated evidence showed that Dicer-independent miRNA and siRNA pathways exist in fungi [39] and that a Dicer-independent miRNA biogenesis pathway, which requires Ago catalysis, exists in vertebrates [40].

The data on significant reduction of amounts of 23–27-nt antisense RNAs corresponding to *suffix* in *ago3* −/− mutant (Table 1) strongly suggest that these fraction of small RNAs corresponds to piRNAs. Taken together the data on RNase protection and analysis of libraries of small RNAs enriched for siRNAs and piRNAs strongly suggest that in ovaries both *suffix* sense and antisense siRNAs are less abundant than *suffix* sense and antisense piRNAs (20 and 80%, respectively). It follows that in ovaries *suffix* transcripts are targeted by both RNAi and Piwi pathways, but piRNAs do most of the work. This conclusion is consistent with the data obtained by the RNase protection assay (Figure 1 and Figure 2).

We observed a decrease in both the antisense and sense siRNAs in the mutants affecting the piRNA pathway (Figure 1 and Figure 2). It is likely that some of these 21 nt long RNAs are piRNAs. Analysis of small RNA libraries revealed that the piRNA length distribution spans the 21 nt region, and a minor portion of piRNAs correspond to this region [12].

### Are the 31 to 54 nt small RNAs the partial digestion products of RNAi and Piwi silencing or evidence for a novel RNA-silencing pathway?

During the last few years, data related to the separation of small RNAs have usually been illustrated with photographs that show the rather narrow region of the gels in which siRNAs, miRNAs and piRNAs are separated. It seems likely that after Zamore et al. [41] described the ladder of 5′ cleavage products of RNAi generated *in vitro,* there was little interest in the longer RNA molecules formed by RNAi-related mechanisms. When we first observed nuclease-protected RNA bands that were >31 nt, we surmised that these longer RNAs were intermediates in the RNAi and Piwi silencing pathways. In fact, this may be true for the bands in the 40–44 nt region of the gel, where longer RNAs are separated (Figure 2); these bands probably correspond to undigested pairs of siRNAs. However, Dicer digests long dsRNAs into double-stranded siRNAs; therefore, equal amounts of both strands should be present not only in the mature siRNAs, but also in the partially cleaved dicing products. The data in Figure 1 do not support the idea that there were substantial levels of incomplete dicing products in the RNA preparations tested or that the RNase digestion in our RNase protection experiments was incomplete. In turn, this indicates that the longer RNA bands corresponding to the *suffix* sense strand that were observed in the gel above the piRNA bands (Figure 2) do not correspond to incomplete dicing products. At present, we cannot explain the reproducible bands that are detected in the 31–54 nt region of the gels. These longer RNA bands were observed in all homozygous mutants tested; among these mutants, one (the *mael* mutant) has a damaged *Drosophila* spindle-class gene that affects all known RNA-silencing pathways [42]. We observed a slight decrease of this class of *suffix* sense small RNAs in the homozygous *mael* mutant, and the mutation had no effect on the siRNA content.

The data related to the longer RNAs supports the hypothesis that there are unknown RNA-regulation pathways that act on longer RNA molecules. Nevertheless, a more detailed analysis of longer RNAs is needed before final conclusions can be drawn about whether they are candidates for a new classes of small RNAs involved in RNA-silencing mechanisms.

*Suffix* corresponds to the 3′ end of the *Drosophila* LINE – F element. The *suffix* region on the 3′ end of the F element is a hot spot in the production of transposable element-specific piRNAs [10]. Because *suffix* is more actively transcribed than its cognate LINE [2], the major portion of the corresponding piRNAs should come from this element. Accumulated evidence has revealed that piRNAs that originate from transposable elements or 3′-ends of mRNAs may have regulatory roles. Recently, Robine et al. reported that the 3′ untranslated regions of an extensive set of mRNAs are processed into piRNAs in *Drosophila* ovaries, murine testes, and *Xenopus* eggs and that their biogenesis depends on primary piRNA components but not on ping-pong components [43]. Untranslated regions of the *Drosophila traffic jam* gene also produce sense piRNAs [44]. It also is of interest that piRNAs produced from two transposable elements target a specific region in the *nos* 3′ untranslated region [45]. These data clearly are consistent with the hypothesis that a concerted silencing mechanism in gene regulation exists, suggesting that small RNAs can simultaneously target different mRNAs [2], [29].

## Materials and Methods

### *Drosophila* strains

*spindle-E −/−* flies were obtained by crossing *ru^1^ st^1^ spn-E^1^ e^1^ ca^1^*/*TM3*, *Sb^1^ e^s^* and *ru^1^ st^1^ spn-E^hls3987^ e^1^ ca^1^*/*TM3*, *Sb^1^ e^s^* mutants, which have a point mutation in the helicase domain of Spn-E and in the P-element insertion into *spn-E*, respectively [31], [32]. *aubergine −/−* flies were obtained by crossing *aub^QC42^/CyO* and *aub^HN^/CyO* mutants [46]. *piwi −/−* flies were obtained by crossing *piwi^2^* and *piwi^3^* mutants, which have a P-*ry11* transposon insertion and a PZ insertional mutation, respectively [47], [48]. We also used *ago2^414^/ago2^414^* [49], *dcr-2^L811Fsx^*/*dcr-2^L811Fsx^* and *mael^r20^/mael^r20^* [50] homozygous flies.

### Cloning procedures

A 77-bp region of *suffix* was cloned into the vectors pGEM-1 and pGEM-2 (Promega) as follows. First, this region was amplified by PCR from a cloned *suffix* copy using the following primers: 5′ cccAAGCTTCACACGCACCCCACC 3′ and 5′ cccgaattCCCTTTCGCCGGAGACGGGAA 3′ (artificial restriction site is shown in lowercase). The amplified product was then digested by *EcoR*I and *Hind*III and cloned into the pGEM-1 and pGEM-2 vectors.

### Detection of suffix-specific small RNAs by an RNase protection assay

Total RNA was isolated from *Drosophila* ovaries, Schneider 2 cultured cells and *Drosophila* wild-type pupae using Trizol reagent (Invitrogen) according to the manufacturer's instructions. pGEM-1 or pGEM-2 plasmids containing the same 77-bp sequence from *suffix* were digested completely with *Hind*III or *EcoR*I and used as templates for the synthesis of strand-specific [^32^P]-labeled RNA probes. Next, 91- or 89-nt-long [^32^P]-labeled RNA (sense or antisense *suffix* RNA, respectively; see Figure S1), was synthesized in 20-µL reactions containing 1 µg of DNA template, 40 mM Tris-HCl (pH 7.5), 6 mM MgCl_2_, 2 mM spermidine, 10 mM NaCl, 10 mM DTT, 1 u/µL RNasin, ATP, GTP and CTP (500 mM each), 0.75 µM [α-^32^P]-UTP (6000 Ci/mmol, EIMB), 10 µM unlabeled UTP and 20 u T7 RNA polymerase (Fermentas).

The mirVana miRNA detection kit (Ambion) was used for the purification of the [^32^P]-labeled RNA probes, for hybridization and for RNase treatment. The [^32^P]-labeled RNA probes were gel-purified by separation on 52-cm long denaturing 12% polyacrylamide gels, 0.2 mm thick, to isolate the full-length 91- or 89-nt-long RNA species and to remove shorter fragments. About 2–5 µg of total *Drosophila* RNA were mixed with about 50,000 cpm of labeled RNA in a 20-µL hybridization mixture (mirVana miRNA detection kit, Ambion), heated for 3 min at 100°C and hybridized at 42°C for 16 h. After hybridization, the samples were treated with RNase A/RNase T1 solution according to the manufacturer's instructions. RNase dilutions were determined experimentally in preliminary experiments to ensure complete removal of non-hybridized [^32^P]-labeled RNA; we used an RNase concentration that slightly affected the protected RNA in order to make sure that non-protected RNA was digested completely. The protected RNA fragments were dissolved in a 5-µL solution containing 90% formamide, 20 mM EDTA and dyes. The probes were separated at 62°C using 12% denaturing polyacrylamide gels that were 0.2 mm thick and 52 cm long. Signals obtained on a phosphorimager were quantified and normalized using rp49 mRNA as an internal reference.

### Detection of suffix sense and antisense transcripts by in situ hybridization

*Suffix* strand-specific DIG-labeled RNA probes were transcribed using T7 RNA polymerase. About 1 µg of DNA template was used in a 20-µL transcription reaction mixture as described above, except that the reaction also contained ATP, GTP, and CTP (1 mM each), 0.65 mM UTP and 0.35 mM DIG-11-UTP (Roche). These RNA probes were dissolved in 20 µL of water plus 80 µL of hybridization solution (HS) containing 50% formamide, 5xSSC, 0.1% Tween 20, 200 µg/mL sheared and denatured salmon DNA and 50 µg/mL heparin. *Drosophila* ovaries were dissected in PBS, fixed for 20 min in 4% paraformaldehyde in PBS, washed three times for 5 min in PBT (PBS/0.1% Tween 20), treated with a solution of 50 µg of proteinase K/mL in PBS (12 min for ovaries), washed with a solution containing 2 mg/mL glycine in PBT for 2 min and twice for 5 min in PBT, re-fixed for 20 min in 4% paraformaldehyde in PBS and again washed twice for 5 min in PBT. After prehybridization in HS at 60°C for 3 to 5 h, the samples were hybridized overnight at 60°C in 300 to 400 µL of HS containing 1 µg of DIG-labeled RNA.

After hybridization, samples were washed three times for 30 min in HS at 60°C, 15 min in 50% HS in PBT at 60°C, twice for 15 min in 2xSSC/0.1% Tween 20 at 60°C, twice for 15 min in 0.2xSSC-0.1% Tween 20 at 60°C, and twice for 15 min in PBT at room temperature. The samples were then incubated for 1–2 h in PBS/0.3% Triton X-100, followed by incubation for 1 h in PBS/0.3% Triton X-100/3% goat serum (blocking step) and then in the same solution with anti-DIG-alkaline phosphatase antibodies (Roche, 1∶2000) for 1 h. Finally, samples were washed five times for 15 min in the blocking solution and once for 15 min in PBT. For staining, samples were washed for 10 min in alkaline phosphatase buffer containing 100 mM NaCl, 50 mM MgCl_2_, 100 mM Tris, pH 9.5, 0.1% Tween 20, and incubated with 1 mL of buffer containing 20 µL of nitroblue tetrazolium-5-bromo-4-chloro-3-indolylphosphate (NBT/BCIP) stock solution (Roche). Development of the reaction was observed under a light microscope; the reaction was usually stopped after 0.5 to 1 h. Samples were then washed five times for 3 min with PBT and mounted on a slide in 60% glycerol in PBS.

### RT-PCR

RNA was isolated from about 100 *Drosophila* ovaries using Trizol reagent (Invitrogen) according to the manufacturer's instructions as described before [51]. Samples were treated with DNase using a DNA-free kit (Ambion), and approximately 2 µg of total RNA, specific primers and M-MLV reverse transcriptase (Promega) were used to synthesize cDNAs corresponding to sense or antisense *suffix* transcripts according to the manufacturer's instructions. For each set of PCR reactions, one reaction contained the cDNA template (RT^+^) and the same RNA probe without addition of reverse transcriptase (RT^−^) (see Figure S2). The number of PCR cycles varied from 28 to 37. Primers for RT-PCR were selected using the Primer Selection Tool program (http://biotools.umassmed.edu/).

The following primers were used for cDNA synthesis: 5′ CAATCTTCTTGTATAAGAACTAACAATAA 3′ (for *suffix* or F element sense transcripts) and 5′ TTCGCACGCACCCCAACCACCTAGCGCGAG 3′ (for *suffix* antisense transcripts). For quantitative PCR using cDNAs corresponding to *suffix* sense or antisense transcripts, the following primers were used: 5′ GTCTAATCCAGCTCAGCAGCC 3′ and 5′ TCGCTGGGTTGGTAGGTCCTT 3′. For quantitative PCR using cDNA corresponding to F element antisense transcripts, the following primer was used: 5′ CACAATCAAAGATTCTGAG 3′. For quantitative PCR using cDNAs corresponding to F element antisense transcripts, the following primers were used: 5′ ATCACTGGGGCACCGTGGTA 3′ and 5′ TCGCTGGGTTGGTAGGTCCTT 3′. The equal efficiencies of different primer pairs used for quantitative PCR for amplification of transcripts corresponding to the F element and *suffix* were confirmed by real-time PCR using the Applied Biosystems 7500 Real-Time PCR System.

The conditions for linear PCR for each set of primers were determined in preliminary experiments using the Mastercycler® personal (Eppendorf). The PCR products were separated in mixed 1% agarose-2% Nu-Sieve agarose gels, and the separation data were evaluated using Quantity One quantitation software (Bio-Rad). Statistical analysis of the fractionated DNA fragments obtained in five independent experiments was performed using Origin software (OriginLab). The identity of amplified DNA fragments was confirmed by sequencing. In preliminary experiments, we performed PCR in duplicate using a radioactive label and obtained similar results.

## Supporting Information

Figure S1 **Sequences of** ***suffix*** **used for synthesis of sense and antisense RNA probes used in the RNase protection experiments.** The same fragment of *suffix* was used for synthesis of [^32^P]-labeled RNAs that make up the *suffix* sense or antisense strands, respectively. The sequences from the T7 promoter or polylinker are shown in lowercase. The pGEM-1 and pGEM-2 vectors containing short polylinker stretches were used to minimize the non-*suffix* sequences in the RNA probes.
(DOC)

Figure S2 **Control for DNA contamination in the RT-PCR experiments.** RT-PCR was performed (37 cycles of amplification) using rp49 primers and RNA isolated from mutant *Drosophila* lines as templates. About 2 µg of the isolated RNA was treated with DNase using a DNA-free kit (Ambion) and then used for cDNA synthesis. Reverse transcription was performed using a specific primer and M-MLV reverse transcriptase (Promega) according to the manufacturer's instructions. For each set of PCR reactions, the cDNA template (RT^+^) and the same RNA probe without addition of reverse transcriptase (RT^−^) were used. DNA contaminating the RNA samples was digested to levels below the PCR detection limits in the conditions used. This supports the idea that the data in Figure 4 are not due to contaminating DNA.
(EPS)

Figure S3 **Validation of the RNase protection assay using chemically synthesized RNAs.** 21, 25 and 27 nt long RNAs corresponding to the sense *suffix* RNA were phosphorylated by T4 polynucleotide kinase using unlabeled ATP. After annealing of RNAs with 89-nt long [^32^P]-labeled *suffix* antisense RNA and treatment with RNases (see Materials and Methods) the RNAse-resistent fragments of antisense RNA were visualized by fractionation in 12% denaturing polyacrylamide gel. D – decade RNA marker (Ambion). M - RNA marker, corresponding to RNA synthesized by T7 RNA polymerase on pGEM-1 plasmid templates digested by *Sma*I endonuclease. The data demonstrate that the length of protected [^32^P]-labeled *suffix* antisense RNA fragments corresponds to the expected after hybridization with the RNAs used.
(EPS)

Figure S4 ***Drosophila*** **ovaries contain** ***suffix*** **sense 26–29 nt long piRNAs.** The gel presented in Figure 2 is shown here after a longer exposure. The aim was to visulize 26–29 nt long piRNAs corresponding to *suffix* sense transcripts that might be present at lower levels due to very strong RNase treatments. The bracket marks RNA bands in the 23–29 nt region that correspond to *suffix* sense piRNAs.
(EPS)
