# Supplementary material for: Both piRNA and siRNA Pathways Are Silencing Transcripts of the Suffix Element in the Drosophila melanogaster Germline and Somatic Cells
Source: PLoS One. 2011 Jul 14;6(7):e21882. doi: 10.1371/journal.pone.0021882 (PMC3136478; doi:10.1371/journal.pone.0021882)
Supplement: Figure S1 — Sequences of suffix used for synthesis of sense and antisense RNA probes used in the RNase protection experiments. The same fragment of suffix was used for synthesis of [32P]-labeled RNAs that make up the suffix sense or antisense strands, respectively. The sequences from the T7 promoter or polylinker are shown in lowercase. The pGEM-1 and pGEM-2 vectors containing short polylinker stretches were used to minimize the non-suffix sequences in the RNA probes. (DOC) [file pone.0021882.s001.doc]

pGEM-2-*EcoR*I – synthesis of 91 nt long ^32^P-sense *suffix* strand from T7-promoter:

5’gggagaccggAAGCTTcacACGCACCCCAACCACCTAGCGCGAGGTCTAATCCAGCT

CAGCaGCCGTTCCCGTCTCCGGCGAAAGGAaatt 3’

pGEM-1-*Hind*III – synthesis of 89 nt long ^32^P-antisense *suffix* strand from T7-promoter:

5’ gggagaccgaattTCCTTTCGCCGGAGACGGGAACGGCTGCTGAGCTGGATTAGACC

TCGCGCTAGGTGGTTGGGGTGCGTGTGAAGCT 3’

**Supplementary Figure1**

**Tchurikov**
